# Finger Vein Recognition Based on Personalized Weight Maps

**DOI:** 10.3390/s130912093

**Published:** 2013-09-10

**Authors:** Gongping Yang, Rongyang Xiao, Yilong Yin, Lu Yang

**Affiliations:** School of Computer Science and Technology, Shandong University, Jinan 250101, China; E-Mails: gpyang@sdu.edu.cn (G.Y.); canyueyang@126.com (R.X.); yangluhi@163.com (L.Y.)

**Keywords:** finger vein recognition, binary pattern, Hamming distance, personalized weight map, general framework

## Abstract

Finger vein recognition is a promising biometric recognition technology, which verifies identities via the vein patterns in the fingers. Binary pattern based methods were thoroughly studied in order to cope with the difficulties of extracting the blood vessel network. However, current binary pattern based finger vein matching methods treat every bit of feature codes derived from different image of various individuals as equally important and assign the same weight value to them. In this paper, we propose a finger vein recognition method based on personalized weight maps (PWMs). The different bits have different weight values according to their stabilities in a certain number of training samples from an individual. Firstly we present the concept of PWM, and then propose the finger vein recognition framework, which mainly consists of preprocessing, feature extraction, and matching. Finally, we design extensive experiments to evaluate the effectiveness of our proposal. Experimental results show that PWM achieves not only better performance, but also high robustness and reliability. In addition, PWM can be used as a general framework for binary pattern based recognition.

## 1. Introduction

Biometric recognition refers to the use of distinctive physiological and behavioral characteristics (e.g., fingerprints, face, hand geometry, iris, gait, signature), called biometric identifiers or simply biometrics, for automatically recognizing a person [1,2]. Since it is difficult to misplace, forge or share biometric identifiers, biometric recognition is more reliable than traditional token-based identification methods (e.g., keys or ID cards) or knowledge-based methods (e.g., passwords or PINs). Besides, biometric recognition also promises better security, higher efficiency, and better user experience in many cases. Among various kinds of biometric identifiers, finger vein recognition is a newly emerging biometrics technology. Medical research proves that each finger has a unique vein pattern that can be used for personal verification [3]. Generally, the finger vein recognition demonstrates some advantages over other biometrics methods [4,5]: (1) non-contact: finger vein patterns are not influenced by surface conditions, and it is more acceptable for the users; (2) live body identification: finger vein patterns can only be identified on a live body without fake finger attacks in fingerprint recognition; (3) higher security: finger vein patterns are internal features that are difficult to forge; (4) smaller device size: most finger vein capturing devices are smaller in size as compared to palm vein based verification devices [6]. Like other traits, finger vein recognition is composed of four main steps, including image capturing, pre-processing, feature extraction and matching. In order to better utilize the features from the segmented blood vessel network for recognition, the authors of [7] extract the finger vein pattern from the unclear image with line tracking. By means of fingerprint features, the minutiae features including bifurcation points and ending points are extracted from finger vein images [8], and a modified Hausdorff distance algorithm is provided to evaluate the identification abilities. In [9] a mean curvature method is proposed, which regards the vein image as a geometric shape, reporting an equal error rate of 0.25% with test images from 125 fingers. Due to the optical blurring and skin scattering problems, the finger vein images are not always clear and may show irregular shadings [10]. However, the image enhancement effect of finger vein is not very good when using traditional image enhancement methods such as histogram equalization and median value filtering [11,12]. To restrain the noises and emphasize the finger-vein linear pattern in the finger-vein image, Zhang *et al.* originally performed a multiscale self-adaptive enhancement transform based on the curvelets to the finger-vein image [13]. Yang *et al.* use the Multi-Channel Gabor Filter, Circular Gabor Filter and the Frangi Filter to enhance the quality of the vein image, respectively, and obtain better results [14–16]. Li *et al.* analyze the shortages of Wavelet transformation and propose an image enhancement algorithm based on the Ridgelet transformation [17].

Multi-biometric systems are provided to further boost the performance of finger vein recognition. The work described in [18] exploits finger vein features in local moments, topological structure and statistics, respectively, and a fusion scheme is adopted for decision making. In [19] first a 2-D Gabor filter is used to filter the original finger vein images, and then the phase and direction texture features for combination in a feature level fusion are extracted. A recognition method based on the score-level fusion of finger veins, fingerprints, and finger geometry features is proposed in [20]. Other similar schemes can be seen in [21,22]. Extracting a clear segmented blood vessel network is very important for finger vein recognition [23], and improperly segmented networks may degrade the recognition accuracy to some extent. In order to avoid this problem, some binary pattern based methods are proposed [24–26], whereby the captured finger vein images are enhanced by modified Gaussian high-pass filter and then LBP, LDP [26] or LLBP [25] are applied to extract the binary codes from the enhanced images. The similarity between the extracted and enrolled binary codes is measured by Hamming distance. Based on the binary pattern methods, the vein images are finally changed into different binary code sequences of the same length. The similarity between two vein images is equivalent to the similarity between two corresponding binary code sequences.

Current binary pattern based finger vein matching methods treat every bit of feature binary codes derived from different images of various individuals as equally important and assign the same weight value to them. Taking the LBP feature as an example, generally speaking, there are many samples captured from the same finger and LBP binary codes (abbreviated in LBPCodes) extracted from these samples, respectively. Considering each bit with same location of these LBPCode series, we find some of them are stable, *i.e.*, the bit values are all 1's or 0's. On the contrary, some other bits are unstable, where some LBPCode series take the value of 1's, and the others a value of 0's. Furthermore, the stable bits of one finger vein are different from the others in terms of location and bit value. Motivated by these phenomena, in our previous work [27], a personalized best bit map (PBBM) method was proposed, in which only the most stable bits called the ‘best bits’ are used for recognition. However, in the PBBM method, the ‘unstable bits’ are all discarded without being used. Can these ‘unstable bits’ contribute some useful information, and how to make the most of these bits? These questions remind us to reconsider the binary pattern methods. We claim that the bits with different degrees of stability should have different contributions to the final matching result. Inspired by [28,29], in this paper, we propose a new finger vein recognition method based on a personalized weight map (PWM). Extensive experiments show that PWM can significantly improve recognition performance as well as robustness.

The rest of this paper is organized as follows: Section 2 presents the principles of binary pattern- based finger vein recognition. Section 3 introduces the definition of the PWM. Section 4 describes the framework of finger vein recognition based on a PWM. Section 5 reports the experimental results to verify the proposed method. Finally, Section 6 concludes this paper.

## 2. Binary Pattern Based Finger Vein Recognition

Binary pattern based finger vein recognition methods aim to solve the low quality images matching problem. Firstly, the captured finger vein images are preprocessed, mainly by ROI extraction, image enhancement, and size normalization. Then certain binary patterns such as LBP, LLBP, or LDP *etc.* are applied to extract features (called binary codes) from the preprocessed images. Finally, the similarity between the extracted and enrolled binary codes is measured by Hamming distance.

### 2.1. Binary Codes by LBP Operator

**Figure 1.**
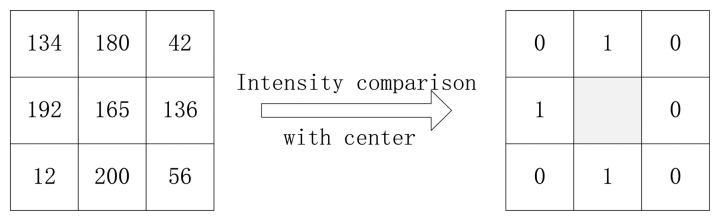
Example of an LBP operator.

Local binary pattern (LBP) is a popular technique used for image representation and classification. LBP has been widely applied in various applications due to its high discriminative power and tolerance to illumination changes such as texture analysis and object recognition. In [30,31] a LBP operator as a nonparametric 3 × 3 kernel for texture classification was proposed. In [26] a LBP-based finger vein recognition method was proposed. A LBP can be defined as an ordered set of binary values determined by comparing the gray values of a center pixel and its neighboring pixels. In other words, first we compare the gray values of the center pixel and its neighboring pixels in a 3 × 3 kernel. If the gray value of a neighboring pixel is bigger than it of the center pixel, a 1 will be given to the corresponding bit in binary code, otherwise, 0 will be given. Then we order these binary codes from random bits clockwise or anticlockwise. We give an example in Figure 1. The binary values can be expressed in decimal form as shown in Equation (1) [26]:

(1) $$LBP(x_{c},y_{c})=\overset{\text{n}=7}{\underset{\text{n}=0}{\text{∑}}}\text{s}({\text{i}}_{n}-i_{c})2^{n}$$

where *i_c_* and *i_n_* in Equation (1) denote the gray value of the center pixel (x_c_,y_c_) and its eight neighboring pixels, respectively. The function s(x) is defined as [30]:

(2) $$s(x)=\{\begin{array}{lll} 1 & if & x\geq 0\\ 0 & if & x<0 \end{array}$$

### 2.2. Binary Codes by LLBP Operator

**Figure 2.**
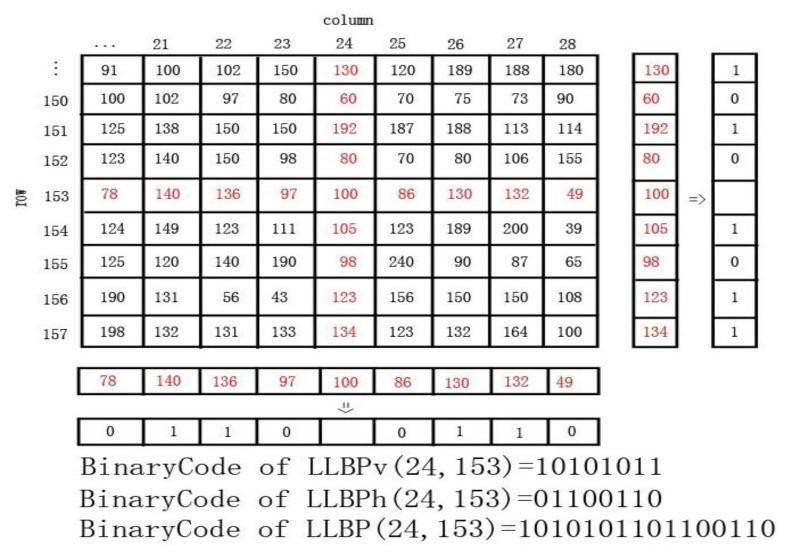
Example of an LLBP operator.

Motivated by LBP, Petpon and Srisuk [32] proposed an LLBP operator for face recognition. The LLBP algorithm first obtains the line binary code along with horizontal and vertical direction separately and its magnitude, which characterizes the change in image intensity such as edges and corners, is then computed. It can be mathematically expressed as in Equation (3)–(5):

(3) $${\text{LLBP}}_{hN,c}(x,y)=\overset{c-1}{\underset{n=1}{\text{∑}}}s(h_{n}-h_{c})\cdot 2^{c-n-1}+\overset{N}{\underset{n=c+1}{\text{∑}}}s(h_{n}-h_{c})\cdot 2^{n-c-1}$$

(4) $${\text{LLBP}}_{vN,c}(x,y)=\overset{c-1}{\underset{n=1}{\text{∑}}}s(v_{n}-v_{c})\cdot 2^{c-n-1}+\overset{N}{\underset{n=c+1}{\text{∑}}}s(v_{n}-v_{c})\cdot 2^{n-c-1}$$

(5) $${\text{LLBP}}_{m}=\sqrt{{\text{LLBP}}_{h}^{2}+{\text{LLBP}}_{v}^{2}}$$

where LLBP_h_, LLBP_v_, LLBP_m_ are LLBP on horizontal direction, vertical direction, and its magnitude respectively, N is the length of the line in pixel, c = (N/2) is the position of the center pixel *h_c_* on the horizontal line and *v_c_* on the vertical line, *h_n_* is the pixel along with the horizontal line and *v_n_* is the pixel along with the vertical line, and ***s*** function is same as in LBP (2). The illustration of LLBP operator is shown in Figure 2.

Employing (2) and (3), the horizontal component of LLBP (LLBP_h_) extracts a binary code of N-1 bits for each pixel. The same numbers of bits are extracted by the vertical component of LLBP (LLBP_v_) using (2) and (4). Consequently, by concatenating the binary codes from LLBP_h_ and LLBP_v_, the total binary code of LLBP for each pixel is 2(N-1) bits.

### 2.3. Matching for Binary Codes

Denoting the binary feature of enrolled image by BinaryCodeA and the binary feature of input image by BinaryCodeB, the Hamming Distance (HD) is generally adopted to measure dissimilarities between the two binary patterns, which are represented as follows:

(6) $$HD=\frac{\left\|\text{BinaryCodeA}⊗\text{BinaryCodeB}\right\|}{\text{LengthofbinaryCode}}$$

In Equation (6), is a Boolean exclusive-OR operator between two binary patterns.

## 3. Personalized Weight Map

**Figure 3.**
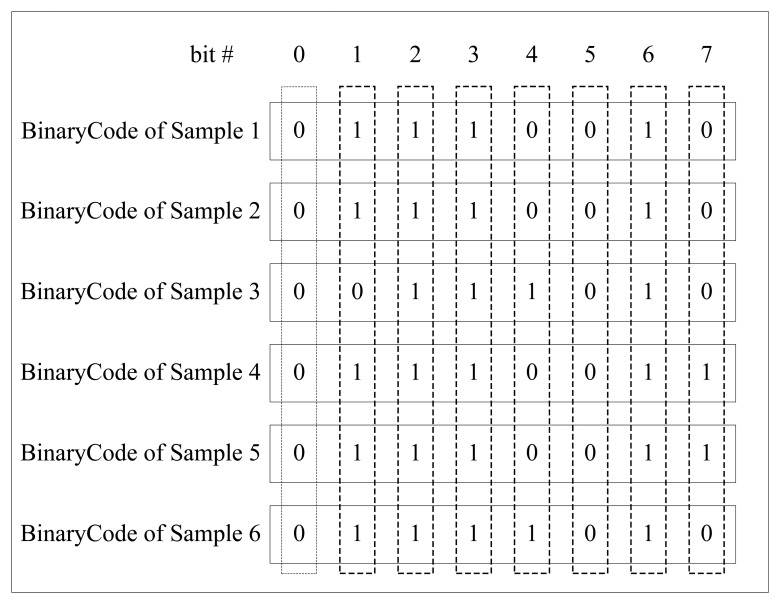
Examples of binary code.

If many samples are captured from a specific finger, we can get the corresponding BinaryCodes by extracting binary features from each sample. As previously mentioned, the values of the bits in the same location of BinaryCodes have different traits. The values of some bits are consistent, either 1's or 0's; the values of some bits have a majority of 1's or 0's; the values of some bits interlace 1's and 0's. Figure 3 gives an example of BinaryCodes of six samples from a certain individual. Apparently, bit 0, bit 2, bit 3, bit 5, bit 6 are very stable, the values of bit 1 are mostly 1's, the values of bit 7 are mainly 0's, and bit4 has interlaced 1's and 0's values.

These investigations provide a valuable clue to improve finger vein recognition performance: the stable bits should play a more important role in matching than the unstable bits, and the stability of a certain bit should be measured to use as a weight for matching. Suppose there are *k* training finger vein images in a finger vein class, and their binary codes are denoted by *code_1_* … *code_k_*, we make k × k times of intraclass matching using these k codes and obtain the average matching result by Equation (7):

(7) $$P=\frac{1}{k\times k}\overset{k}{\underset{a=1}{\text{∑}}}\overset{k}{\underset{b=1}{\text{∑}}}{\text{code}}_{a}⊙{\text{code}}_{b},P=\{p_{1}\ldots ,p_{n}\}$$

where *P* is a vector with the same length as the binary codes, and ⊙ means logical NOT of ⊕in Boolean operations. For the *ith* bit, *p_i_* represents the probability of the matching result being 1. Every *p_i_* is of different value and bigger *p_i_* indicates that bit *i* is more stable. Suppose there are k binary codes in the same class, and the value of a certain bit *i* is 1's for *m_1_* times and 0's for *m_0_* times (*m_1_* + *m_0_* = *k*), so the average result of bit *i* is $P_{i}=\frac{m_{1}^{2}+m_{0}^{2}}{{\left(m_{1}+m_{0}\right)}^{2}}$ by Equation (7). It is obvious that *p_i_* is always between 0.5 and 1. For convenience, we normalize it to [0, 1] by Equation (8).

(8) W=2P−1

where W (W = {*w_1_*; … ; *w_n_*}) is called the “weight map”. Because the weight map is different from individual to individual, we call the weight map for each individual the Personalized Weight Map (PWM).

With PWM, our improvement to the traditional Hamming distance is that a “weight map” *W_A_* of the registered finger vein class A, which is denoted by an n-dimensional vector {*w_1_*; *w_2_*; …; *w_n_*}^T^, is introduced to the matching function as follows:

(9) $${\text{Similarity}}_{AB}=1-\frac{\left\|(\text{BinarycodeA}⊗\text{BinarycodeB})\times W_{A}\right\|}{\left\|W_{A}\right\|}$$

If *Similarity_AB_* is bigger more than a given threshold, the two finger vein images are considered from the same individual.

## 4. The Proposed Method

**Figure 4.**
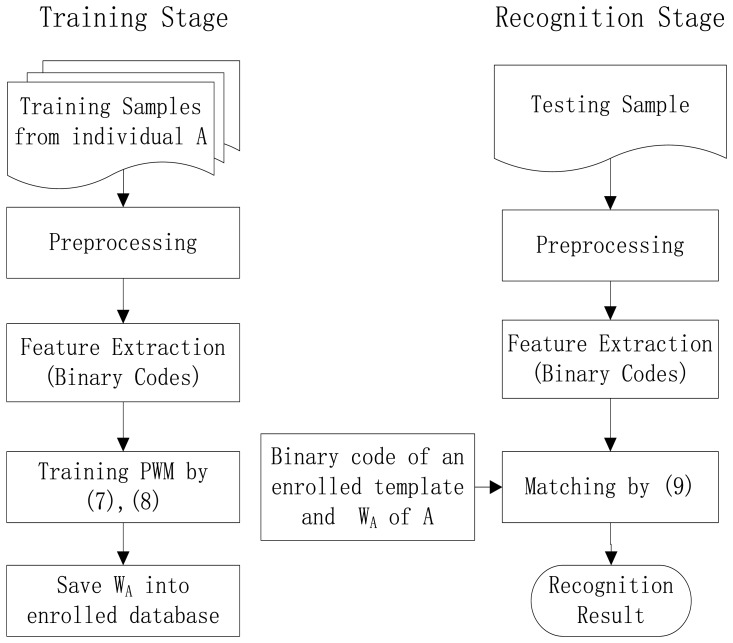
Framework of the proposed method.

Based on the mentioned PWM definition, we propose a finger vein recognition method which mainly involves two stages: a training stage and a recognition stage. The training stage aims to generate the PWM for each individual, which includes preprocessing, feature extraction and finally generating the PWM using certain number of training samples. In the recognition stage, we first preprocess the test sample, and then extract the corresponding binary code, and next compute the similarity of binary codes between this test sample and the template of a certain individual in the enrolled database, finally obtain a recognition result with a given threshold. The framework of the proposed method is demonstrated in Figure 4.

### 4.1. Preprocessing

**Figure 5.**
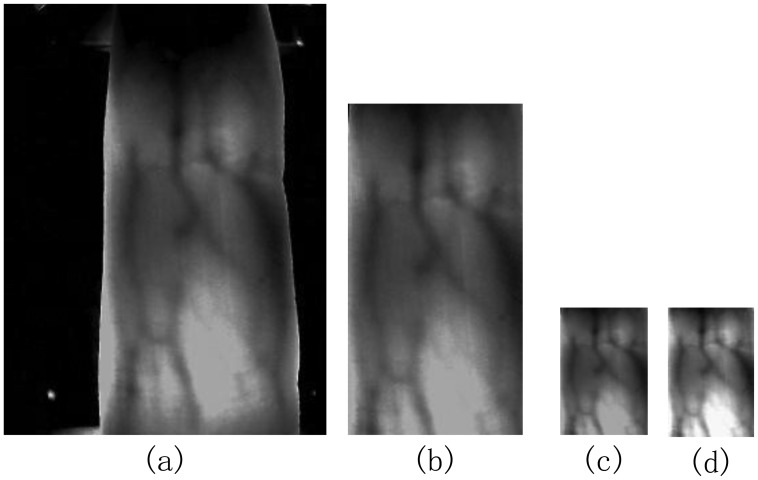
Examples of preprocessing. (**a**) Original image. (**b**) ROI extraction. (**c**) Size normalization. (**d**) Gray normalization.

Like in reference [27], the preprocessing operation mainly refers to ROI extraction, size normalization and gray normalization. Figure 5 shows the results in each step.

### 4.2. Feature Extraction

The feature extraction process mainly involves how to extract the binary code of a certain finger vein image by a given binary pattern operator such as LBP, LLBP or LDP, *etc.* It should be pointed out that the PWM method can be regarded as a general framework, and then we call a certain binary pattern such as LBP the base feature of the PWM.

### 4.3. Training Process

Since each individual has his/her own PWM, so we need to train the corresponding PWM for each individual, and apparently a certain number of training samples from each individual is needed. First we get the corresponding BinaryCodes by extracting binary features from each training sample of one individual. Then the PWM for this individual will be computed by Equations (7) and (8). With different numbers of training samples, the corresponding PWMs are different too. Although the number of training samples is the same, the corresponding PWM is also different for different individuals. Considering the above situation, the main objective of the training process is to generate the optimal PWM for each individual. Several factors should be explored such as: (1) how many samples are suitable to train the PWM? (2) The robustness of the training method. Given the condition that the number of training samples is fixed, if the samples are different, can the recognition performance be kept stable? (3) With an increasing number of training samples, can the recognition performance also be boosted?

### 4.4. Recognition Process

In the recognition stage, we first extract the corresponding binary code of the test sample, and then compute the similarity of this test sample and the enrolled sample of a certain individual. The recognition process can work in two modes: verification mode and identification mode. In the verification mode, the class of the input finger vein (test sample) is known, and each of the samples is matched with all the other samples in the database. A successful matching is called intra-class matching or genuine, if the two samples are from the same class. Otherwise, the unsuccessful matching is called interclass matching or imposter. In the identification mode, we do not know the class of the input finger vein images. The samples of each class in the enrolled database can be classified into two categories: templates and probes. The probes were matched with all the template models, and for each probe, the matching results were ordered according to the matching scores.

## 5. Experimental Results and Analysis

### 5.1. The Experimental Database

**Figure 6.**
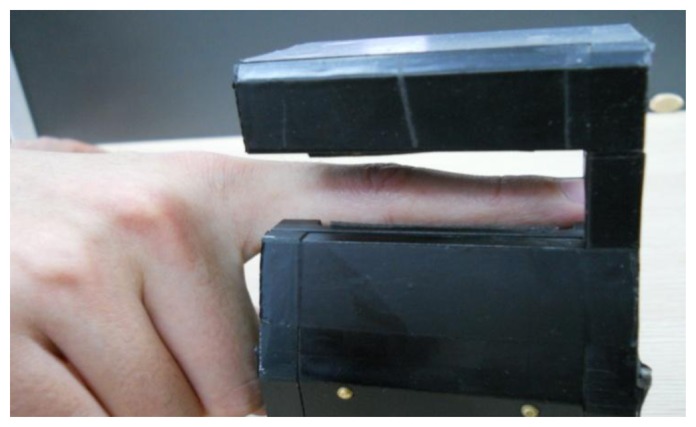
The finger vein image capture device.

The experiments were conducted on our new finger vein database which is collected from 34 individuals, including 20 males and 14 females; they are students, professors and staff in our school. The age of the participants is between 19 and 48 years. We collected the images in two separate sessions separated by 20 days. In each session, the subject was asked to provide 10 images for each of the left index finger, the left middle finger, the right index finger and the right middle finger. The capture device was manufactured by the Joint Lab for Intelligent Computing and Intelligent System of Wuhan University, China. The capture device mainly consists of a near-infrared light source, lens, light filter, and image capture equipment. Vein patterns can be viewed through an image sensor which is sensitive to near-infrared light, because near-infrared light passes through human body tissues and is blocked by pigments such as hemoglobin or melanin. A groove in the shell of the device is used to guide the finger orientation, and the capture device is illustrated in Figure 6.

### 5.2. The Experimental Settings

The original spatial resolution of the finger vein image is 320 × 240, After ROI extraction and size normalization, the size of the region used for feature extraction is reduced to 96 × 64. We set the parameter N of LLBP as 21 in our experiment. In order to verify the proposed method comprehensively, we design four experiments: (a) in Experiment 1, we take LBP as base feature to generate the PWM, and evaluate performance of the proposed method in verification mode and identification mode respectively. (b) Experiment 2 reports the recognition result of the PWM, which takes LLBP as base feature. (c) Experiment 3 is conducted to compare the performances of the PWM and the PBBM method which was proposed in our previous work [27]. (d) Experiment 4 discusses the computation time of the PWM method.

### 5.3. Experiment 1—Comparison between LBP and PWM

#### 5.3.1. Verification Mode

In the experiments, we use the first ten samples of each class in the database to generate the PWM and use the other ten as testing samples. In order to avoid class imbalances, we use all the ten testing vein images in intra-class matching meanwhile the first two of the ten testing vein images in interclass matching. Consequently, there are 6,120 (136 × 45) intra-class matching and 73,440 (136 × 135 × 4) interclass matches in total. In this paper, we evaluate the recognition performance by the equal error rate (EER), the false rejection rate (FRR) at 0.1% false acceptance rate (FAR) and the FAR at 0.1% FRR, respectively. The EER is mainly used to measure the overall performance of biometric systems because the FRR and FAR are treated equally. On the other hand, the FRR at 0.1%-FAR is suited for high security systems, as in those systems, false acceptance errors are much more critical than false rejection errors. On the contrary, the FAR at 0.1%-FRR shows the acceptance rate of impostors when no genuine rejections are desired.

**Figure 7.**
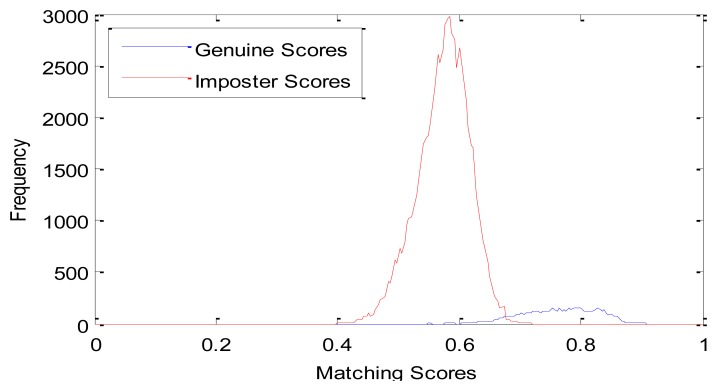
Genuine and imposter matching score distributions by LBP.

**Figure 8.**
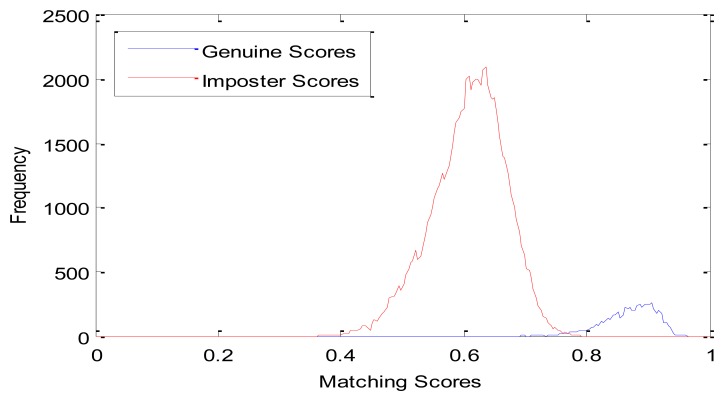
Genuine and imposter matching score distributions by PWM.

We compare the proposed method with the LBP based method described in [26]. Genuine and imposter matching score distributions of these two methods are shown in Figures 7 and 8, respectively. From Figure 7, we can see that the genuine and imposter matching scores by LBP are overlapped between 0.6 and 0.7. Otherwise, the genuine matching scores by PWM are mainly between 0.75 and 0.95, and the imposter matching scores are mainly between 0.40 and 0.75.

**Figure 9.**
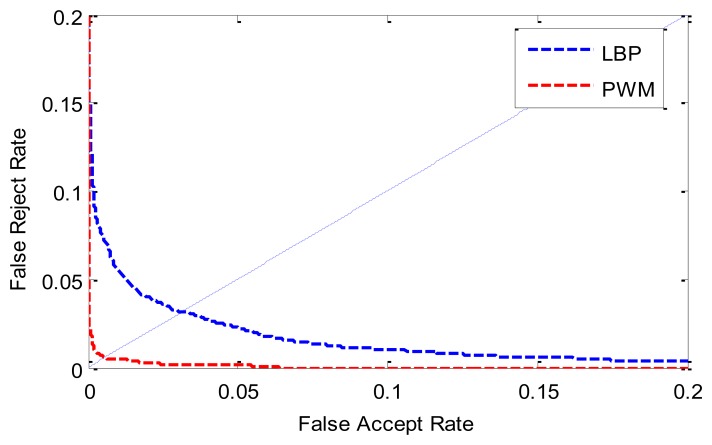
ROC curves by different methods.

The ROC (receiver operating characteristic) curves are shown in Figure 9. Table 1 lists the ERR, FRR at 0.1%-FAR and FAR at 0.1%-FRR values. From Figure 9 and Table 1, we can see that the proposed method achieves a much lower EER than the LBP-based method. This indicates that the stability of a certain bit contributes to the final matching with a different degree. Traditional binary pattern-based finger vein matching methods treat every bit of feature binary codes derived from different image of various individuals as equally important and assign the same weight value to them. Such a uniform matching strategy definitely is not optimal in terms of finger vein recognition accuracy because it ignores significant differences of finger vein texture patterns among individuals and variations of image structures from the same individual.

To verify the robustness of the PWM trained by samples acquired in different sessions, we randomly select ten samples of each class in the database to generate PWM and used the other ten as test samples. We repeat this process ten times, and the statistical data of the verification performance (EER) is shown in Table 2. From Table 2, we can see that the PWM is robust for training and testing samples acquired from different sessions and always has better performance than traditional LBP based method.

#### 5.3.2. Identification Mode

**Figure 10.**
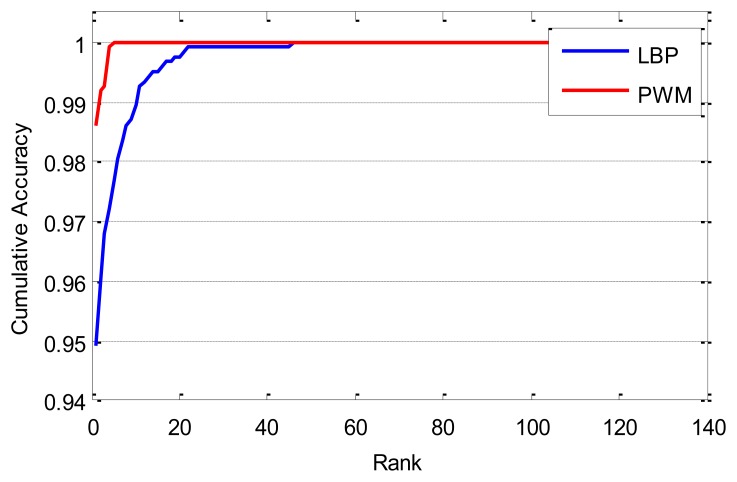
Cumulative match curves by different methods.

The experiments of identification are also reported. We use the first ten samples of each class in the database to generate the PWM and randomly select one from the other ten samples as template; the rest nine samples are taken as probes. Therefore, there are 136 templates, and 1,224 (136 × 9) probes in total. For each probe, there is a corresponding gallery of the same person, the gallery is sorted by decreasing similarity for each probe, and the probe is said to be correctly recognized at rank k if the gallery of the same person is among the first k images in the sorted gallery. Then, we can get the cumulative match curves as shown in Figure 10. The cumulative matching performance, rank-one recognition rate, and lowest rank of perfect recognition (*i.e.*, the lowest rank when the recognition rate reaches 100%) are listed in Table 3. From the experimental results, we can see that the performance of the proposed method is much better than that of the LBP-based method.

#### 5.3.3. On Different Number of Training Samples

According to the PWM definition, the proposed method needs a certain number of samples to generate the PWM for each individual. This experiment is conducted to show the effect on recognition performance when using different number of training samples to generate a PWM. For the sake of justice, we measure the performance by fixing the number of testing samples and adjusting the number of training samples to generate the corresponding PWMs. We set the last six as testing samples of each class in the established database, so that the number of training samples is between 2 and 14 (here we neglect the number of training samples is equal to 1, due to its meaninglessness ).

**Figure 11.**
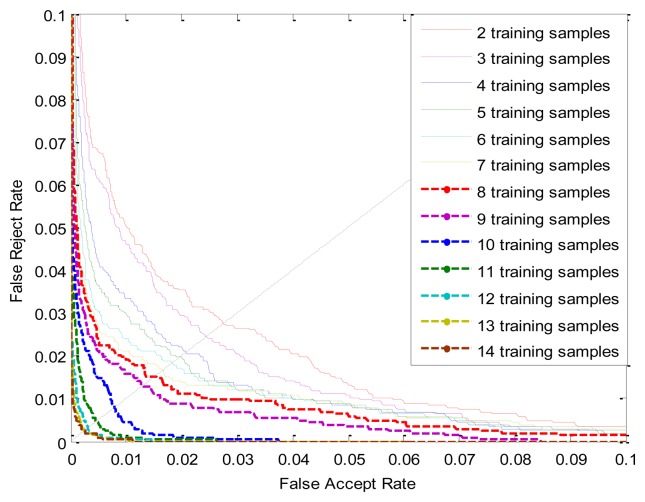
ROC curves by different number of training samples.

The ROC curves of PWMs trained by different number of samples are shown in Figure 11, The ERR, FRR at-0.1%-FAR and FAR at 0.1%-FRR values are listed in Table 4. From Figure 11 and Table 4, we can see that better performance is achieved with an increasing number of training samples; it also indicates that we can only use a small number of training samples of each class to generate a competitive PWM. For example, capturing the ten samples for each finger is not a very tedious task.

**Figure 12.**
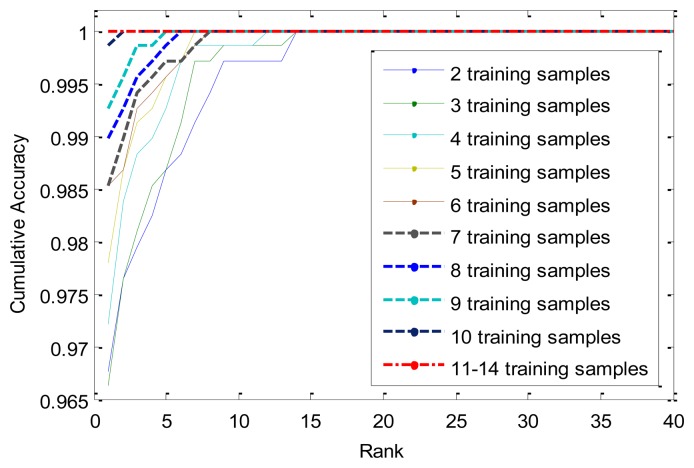
Cumulative match curves by different number of training samples.

We also conduct experiments on the identification mode. Table 5 and Figure 12 show the experimental results. Apparently, when the number of training samples reaches 11, the rank-one recognition rate can achieve 100%, it means that all the probes can be identified correctly.

### 5.4. Experiment 2—Comparison between LLBP and PWM

**Figure 13.**
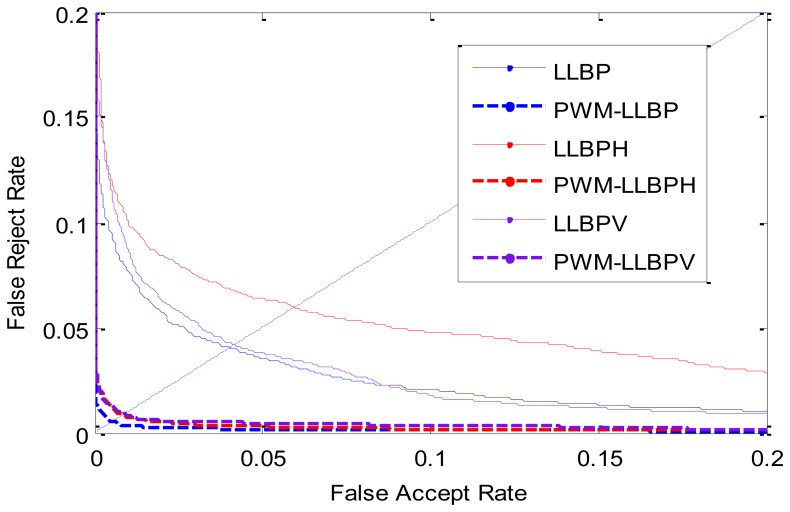
ROC curves by different methods.

With an identical experimental setting in Experiment 1 such as training number and experimental steps, this experiment reports the recognition result by using LLBP as base feature to generate the PWM. The verification results are reported in Table 6, and the ROC curves are illustrated in Figure 13. From Table 6, we can see that the PWMs have better performance than the traditional LLBPs method.

**Figure 14.**
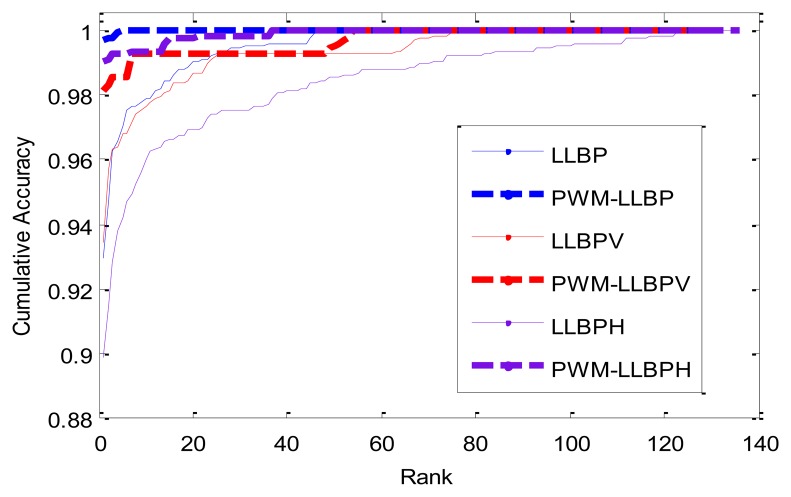
Cumulative match curves by different methods.

The identification results are shown in Table 7, and the cumulative match curves are illustrated in Figure 14. From the experimental results, we can see that the performance of the proposed method is also much better than that of the LLBP-based methods in identification mode. In addition, PWM-LLBP has overall better performance than PWM-LLBP_h_ and PWM-LLBP_v_ due to its combination of vertical and horizontal direction information at same time.

### 5.5. Experiment 3—Comparison between PBBM and PWM

According to the definition of PBBM and PWM, only the ‘best bits’ are used for matching in the PBBM method, whereas in the PWM method all the bits which contribute different weight values are used for matching. The difference between the PWM and PBBM can be described as follows: (1) In essence, the PBBM is a template; the binary code of a test sample matches with the PBBM directly. In addition, one class has only one template; it is difficult to conduct multi-template matching strategy. The PWM is just a weight map; it combines with two binary codes for matching. (2) The PWM has more generality, and the PBBM can be taken as a special case of the PWM. If the best bits of PBBM are very few, the recognition result may be not credible. The PWM utilizes not only the best bits (stable bits) but also other unstable bits, and assigns to these bits different weight values according to their stability.

**Figure 15.**
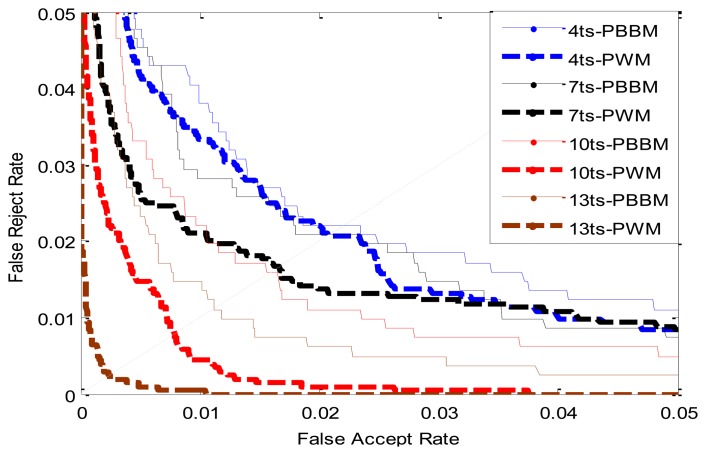
ROC curves by different methods.

This experiment is conducted to compare the recognition performance of PBBM and PWM when using different number of training samples. Using the same experimental setting of the experiment described in section 5.3.3, the last six samples of each class in the established database are fixed as the testing samples, and the number of training samples is changing from 2 to 14. The ERR values of PBBM and PWM are listed in Table 8. The contrast of ROC curves between PBBM and PWM trained by different samples are shown in Figure 15 (in Figure 15, ts is an acronym of training samples). From Table 8 we can see that as increasing the training samples from 3 to 14, the EER values of PWM are always lower than those of PBBM, and the superiority of PWM is more pronounced when the training samples is up to 10. It indicates that the ‘unstable bits’ which are discarded by the PBBM method can contribute to the final matching.

### 5.6. Experiment 4—Measurement of Time Complexity

All the above experiments are implemented in MATLAB, and run on a PC with a 2.4 GHz CPU and 2 G Memory. Average computation times for key operations in our system are listed in Table 9. Similar to the above experimental setting, we use 10 training samples to evaluate the PWM training time which includes the time interval from input of training images to getting the PWM. The matching time interval is from the input of two images to getting the final matching result. Although the average training time is somewhat time-consuming, the training process can be done off-line. In a word, from Table 9, we can see that the proposed method can be used in real time.

### 5.7. Discussion

According to the definition of PWM, given the fixed number of training samples, the possible weight value of a bit in the PWM is also decided. For example, if the number of training samples is 8, then the possible weight values of a certain bit are 0.00, 0.0625, 0.25, 0.5625, or 1.00 respectively. If the number of training samples is only 2, then the possible weight values of a certain bit are 0.00, or 1.00. Apparently we can conclude that the more number of training samples, the better of recognition performance. This fact also was verified in Experiment 1.

**Figure 16.**
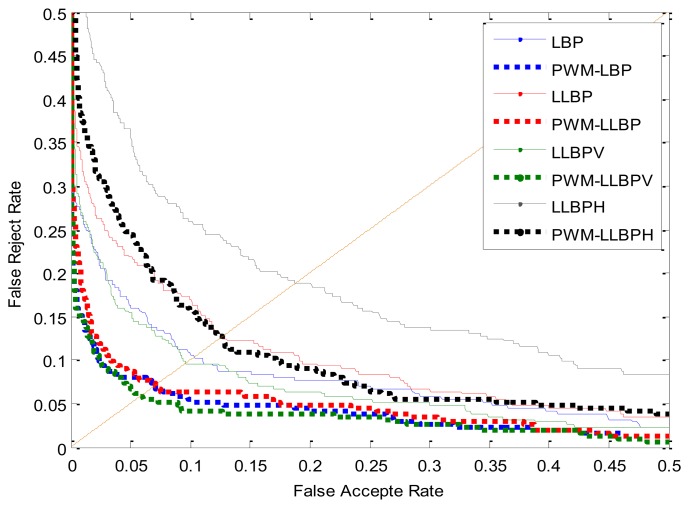
ROC curves by different methods.

There is another open finger vein image database from Hong Kong Polytechnic University [33], which consists of 3,132 finger vein images taken from 156 volunteers over an average interval of 66.8 days. Among these 156 volunteers, only 105 subjects turned up for the imaging during the second session, other subjects contribute six images in the first session. Like in the analysis above, it's a limitation of the performance advantage of the PWM method with fewer training images, but in order to further assess the performance of the proposed method, we also conduct a simple experiment on this database. In this part, we use the first four samples of each class in the database to generate the PWM and the last two as testing samples. The verification results are reported in Table 10 and the ROC curves are illustrated in Figure 16. We get the region of interesting (ROI) images via the method proposed in [34] and extract features such as LBP and LLBP directly, without performing further image enhancement, so the EERS gained in this database are all higher than that in our database. From Table 10, we can see that the PWM-method has better performance than the Base-method, and we ascertain that a much better performance will gained as increasing the training samples.

## 6. Conclusions

This paper presents a novel finger vein recognition method based on the concept of a personalized weight map. The experimental results show the superior performance of our method in comparison with other traditional binary pattern based methods. The advantages of PWM can be summarized as follows: (1) PWM can effectively reduce the adverse effect of unstable bits. Through the training process, different bits are assigned different weight values which are used for final matching. The stable bit has a higher weight value and *vice versa*. (2) PWM must be trained for every individual respectively, so PWMs are different from individual to individual, thus PWMs can be regarded as a kind of personalized feature that reflects the differences between each individuals remarkably. (3) PWM is highly robust and reliable; this has been verified in the aforementioned experiments. (4) PWM can be used as a general framework for binary pattern based recognition. Besides LBP and LLBP, we can use other binary code to generate the corresponding PWM, and this will be the subject of our future work. At last, we expect that the PWM will display better adaptability to finger vein images with low quality.

## Figures and Tables

**Table 1.** Verification performance by different methods.

	**EER**	**FRR at-0.1%-FAR**	**FAR at-0.1%-FRR**
LBP method	0.0316	0.1250	0.5157
PWM method	0.0056	0.0216	0.1150

**Table 2.** The statistical data of EER by random sampling.

	**Minimum**	**Maximum**	**Mean**	**Variance**
LBP method	0.0237	0.0306	0.0273	5.9489e-06
PWM method	0.0041	0.0065	0.0053	7.1540e-07

**Table 3.** Identification performance by different methods.

	**Rank-one Recognition Rate**	**Lowest Rank of Perfect Recognition**
LBP method	94.93%	46
PWM method	98.61%	5

**Table 4.** Verificaion of performance with different numbers of training samples.

	**EER**	**FRR at-0.1%-FAR**	**FAR at-0.1%-FRR**
2 training samples	0.0275	0.1221	0.3175
3 training samples	0.0245	0.1069	0.2526
4 training samples	0.0207	0.0877	0.2663
5 training samples	0.0196	0.0760	0.2468
6 training samples	0.0182	0.0667	0.2410
7 training samples	0.0166	0.0539	0.2414
8 training samples	0.0152	0.0529	0.1604
9 training samples	0.0128	0.0426	0.0702
10 training samples	0.0078	0.0338	0.0185
11 training samples	0.0045	0.0245	0.0098
12 training samples	0.0030	0.0118	0.0056
13 training samples	0.0025	0.0064	0.0048
14 training samples	0.0025	0.0054	0.0048

**Table 5.** Identification of performance with different numbers of training samples.

	**Rank-one Recognition Rate**	**Lowest Rank of Perfect Recognition Rate**
2 training samples	96.76%	14
3 training samples	96.62%	14
4 training samples	97.21%	12
5 training samples	97.79%	7
6 training samples	98.53%	8
7 training samples	98.53%	8
8 training samples	98.97%	6
9 training samples	99.26%	5
10 training samples	99.85%	2
11 training samples	100%	1
12 training samples	100%	1
13 training samples	100%	1
14 training samples	100%	1

**Table 6.** Verification performance by different methods.

	**EER**	**FRR at 0.1%-FAR**	**FAR at 0.1%-FRR**
LLBP	0.0402	0.1426	0.4937
PWM-LLBP	0.0052	0.0176	0.1983

LLBPv	0.0419	0.1946	0.4851
PWM-LLBPv	0.0088	0.0319	0.2013

LLBPh	0.0592	0.1750	0.8041
PWM-LLBPh	0.0085	0.0417	0.1452

**Table 7.** Identification performance by different methods.

	**Rank-one Recognition Rate**	**Lowest Rank of Perfect Recognition**
LLBP	92.97%	50
PWM-LLBP	99.67%	5

LLBPv	93.46%	76
PWM-LLBPv	98.13%	44

LLBPh	89.87%	123
PWM-LLBPh	99.02%	37

**Table 8.** EER contrast with different training samples.

	**EER of PBBM**	**EER of PWM**
2 training samples	0.0270	0.0275
3 training samples	0.0260	0.0245
4 training samples	0.0221	0.0207
5 training samples	0.0220	0.0196
6 training samples	0.0208	0.0182
7 training samples	0.0208	0.0166
8 training samples	0.0196	0.0152
9 training samples	0.0184	0.0128
10 training samples	0.0159	0.0078
11 training samples	0.0148	0.0045
12 training samples	0.0134	0.0030
13 training samples	0.0111	0.0025
14 training samples	0.0122	0.0025

**Table 9.** The average computation times.

**Base Feature**	**Preprocessing**	**Feature Extraction**	**Training PWM**	**Matching**
LBP	43.7 ms	1.3 ms	363.9 ms	90.5 ms
LLBP	43.7 ms	9.2 ms	435.7 ms	106.3 ms
LLBPv	43.7 ms	4.1 ms	412.6 ms	95.7 ms
LLBPh	43.7 ms	3.5 ms	398.5 ms	93.2 ms

**Table 10.** Verification performance by different methods.

	**LBP**	**LLBP**	**LLBPV**	**LLBPH**
Base-EER	0.1022	0.1259	0.0963	0.1891
PWM-EER	0.0701	0.0705	0.0571	0.1232
